# Family-Focused Digital Mental Health Care for Pediatric Oppositional Symptoms and Caregiver Outcomes: Retrospective Analysis

**DOI:** 10.2196/82039

**Published:** 2026-06-22

**Authors:** Darian Lawrence-Sidebottom, Kelsey McAlister, Donna McCutchen, Monika Roots, Jennifer Huberty

**Affiliations:** 1 Bend Health, Inc, 321 East Washington Ave, #200, Madison, WI, 53703, United States, 1 800-516-0975; 2 Fit Minded, Inc, Phoenix, AZ, United States

**Keywords:** oppositional defiant disorder, attention-deficit hyperactivity disorder, parental stress, pediatric mental health, therapy

## Abstract

**Background:**

Oppositional symptoms in youth are characterized by an angry or irritable mood and excessive defiance (eg, arguing), negatively impacting the mental well-being of children, adolescents, and their caregivers. Pediatric digital mental health interventions (DMHIs) that approach care from a whole-family perspective may effectively address mental health (MH) symptoms in both pediatric participants and their caregivers, though this has not been explored in the context of oppositional symptoms.

**Objective:**

The purpose of this study was to assess oppositional symptoms in children and adolescents (aged 6 to 17 years) participating in care within the real-world conditions of a family-centered DMHI. We aimed to (1) examine baseline oppositional severity and its associations with child demographic and clinical characteristics (eg, co-occurring MH symptoms), and caregiver symptoms; (2) evaluate demographic, clinical, and engagement factors associated with oppositional symptoms during care with the DMHI; and (3) determine whether changes in oppositional symptoms during care are associated with improvements in caregivers’ stress, burnout, and sleep.

**Methods:**

Retrospective analyses included 3781 child-caregiver pairs who participated in coaching and therapy with Bend Health Inc, a family-centered, pediatric DMHI. Assessments at baseline and monthly during care measured pediatric and caregiver symptoms. Children and adolescents were grouped by oppositional severity at baseline: not significant, subclinical, and clinical. Pediatric characteristics, care type, and caregiver symptoms were compared between groups. Linear mixed-effects models assessed oppositional symptoms over months and then tested whether oppositional severity and rate of symptom improvement were associated with caregiver outcomes over time.

**Results:**

Baseline oppositional symptoms were not significant for 51.55% (1949/3781), subclinical for 26.47% (1001/3781), and clinical for 21.98% (831/3781). More severe oppositional symptoms were associated with younger age (*P*<.001), nonfemale sex (*P*<.001), White race or ethnicity (*P*<.001), higher rates of MH diagnoses (all *P*<.001), and higher rates of co-occurring inattention, hyperactivity, depression, and sleep problems (all *P*<.001). Odds of elevated caregiver symptoms increased with more severe oppositional symptoms (all *P*<.001). At the end of care (final follow-up), oppositional symptoms improved for 73.93% (740/1001) with subclinical symptoms and 82.43% (685/831) with clinical symptoms. Symptom trajectories followed a logarithmic curve, with the greatest improvements in the first several months (*P*<.001). While more severe oppositional symptoms were associated with more severe caregiver stress, burnout, and sleep problems (all *P*<.001), monthly improvements in caregiver symptoms were significantly larger for those whose child improved more quickly (all *P*<.001).

**Conclusions:**

Family-centered DMHIs may effectively address pediatric oppositional symptoms, as well as co-occurring impairments in caregiver well-being. These findings highlight the broader, system-level impact of scalable DMHIs (such as Bend) in addressing complex family MH needs. Future work should examine these effects in the long term and evaluate opposition-specific care pathways within DMHIs.

## Introduction

Oppositional and defiant behaviors in children and adolescents are characterized by an angry and irritable mood, temper tantrums, frequent arguing, and vindictiveness [1]. Emerging as early as 4 years of age [2], oppositional symptoms may become persistent and severe enough to meet the criteria for oppositional defiant disorder (ODD) [3]. Estimates of the prevalence of ODD vary widely [1], with one meta-analysis of 25 studies citing a 3.3% global prevalence [4], and another study reporting a 1 in 10 lifetime prevalence in the United States [5]. These rates are even higher among clinical populations and youth in the justice system [6,7]. ODD is also a known precursor to more severe behavioral diagnoses, including conduct disorder (CD) and antisocial personality disorder [8-11], and is associated with early disciplinary issues [11], elevated risk for substance use, and higher prevalence of co-occurring mental health (MH) challenges [2,5,6,10-12]. Attention-deficit hyperactivity disorder (ADHD), a neurodevelopmental disorder characterized by disruptions in attention and executive function, is also closely linked to ODD, as these disorders share overlapping risk factors and approximately 60% of individuals with ADHD also have ODD [12].

While various environmental and genetic factors appear to be associated with ODD, maladaptive family dynamics are particularly predictive of the development of ODD [13,14]. Disruptions in the parent-child relationship, including inconsistent or reactive discipline, can inadvertently reinforce defiant behaviors, contributing to a cycle of escalating conflict and emotional strain within the family [13]. In a study of preadolescent boys (aged 8 to 13 years), negative reinforcement of oppositional and defiant behaviors was associated with a higher incidence of antisocial behavior over the following 2 years [14]. Caregivers are also negatively affected by this cycle, as persistent child behavior problems can erode caregivers’ emotional regulation and capacity to implement effective strategies, further intensifying strain within the household. Caregivers of children with ODD often report high levels of stress [15-18], potentially related to their child’s disciplinary issues, school difficulties, and, in some cases, involvement with the justice system [14,19]. They also experience elevated rates of sleep disturbances, anxiety, depression, and substance use [7,20-22]. Together, these patterns highlight the urgent need for accessible, family-centered interventions that can interrupt problematic dynamics and promote healthier outcomes for both children and their caregivers.

Parent management training and family-based interventions are considered effective ODD treatments for children, especially those aged 12 years and younger [1,23,24]. Children who have developed the skills to reflect on their emotions and behaviors (around 8 years of age) can have a more active role in treatment and, therefore, may also benefit from cognitive behavioral therapy (CBT) and social problem-solving skill training [24-27]. However, there is some evidence suggesting that oppositional symptoms are relatively resistant to treatment, especially those that do not involve caregiver interventions. Studies among youth have demonstrated stable or worsening symptoms over time, even as comorbid MH symptoms improve, especially in the absence of targeted intervention [7,28,29]. These challenges have led to growing interest in integrated approaches that combine accessible parent-focused training with child-directed behavioral care (eg, CBT), offering a more comprehensive, whole-family model that may be better suited to address both child symptoms and broader family dynamics.

Digital mental health interventions (DMHIs) address youth MH challenges by providing timely access to care, supporting individualized treatment plans, and integrating multiple types of care within one platform [30-33]. Studies have demonstrated the efficacy of DMHIs for many different symptom domains in children and adolescents, with most studies focusing on anxiety, depression, and stress [31,33-37]. While several coaching and therapy-based DMHIs treat child behavior problems, evaluations often overlook oppositional behaviors. Instead, many programs rely on broad checklists (eg, the pediatric symptom checklist) to measure general progress [37], or claims of symptom improvement are not peer-reviewed [38]. While one study of symptoms during coaching and therapy with a family-centered pediatric DMHI (Bend Health, Inc) used an opposition-specific measure, findings of improvement were limited by a relatively short treatment duration (<2 months) and small sample size (n=20) [28]. This study also did not address the role of co-occurring symptoms (eg, anxiety or depression) or program engagement in treatment outcomes. Further, while there is evidence that caregiver well-being improves during a child’s participation with various pediatric DMHIs [21,22,39,40], and oppositional symptom reduction mediates improvements in caregiver well-being [41], the extent to which child and adolescent oppositional symptoms may associate with caregiver outcomes during care with a DMHI has not been addressed. There remains a need for a large-scale evaluation of paired youth-caregiver outcomes within the context of a real-world DMHI using a family-centered approach to address oppositional symptoms.

The purpose of this retrospective analysis was to assess oppositional symptoms in children and adolescents (aged 6 to 17 years) participating in care with a family-centered DMHI. We aimed to (1) examine baseline oppositional symptom severity and its associations with child demographic and clinical characteristics (eg, co-occurring MH symptoms), and caregiver symptoms; (2) evaluate demographic, clinical, and engagement factors associated with oppositional symptoms during care with the DMHI; and (3) determine whether changes in oppositional symptoms during care are associated with improvements in caregivers’ stress, sleep, and burnout.

## Methods

### Study Design and Participants

This retrospective analysis included outcomes of pediatric members (aged 6 to 17 years) and their caregivers enrolled in coaching, therapy, or psychiatry programs with Bend Health Inc (Bend) between January 2023 and June 2025. The programs evaluated in this study were offered by Bend Health, Inc, through Bend Health Psychiatric Services and affiliated physician practices. Programs were delivered by MH providers in video-based sessions, and regular symptom assessments facilitated measurement-based care. Children were not eligible to enroll in Bend programs if they were in detox for alcohol or illicit drug use, or had any of the following: active command hallucinations or severe thought disorganization, moderate-to-severe intellectual disability, neurocognitive disorder with severe memory or functioning difficulties, severe eating disorders (including BMI <18), or severe autism spectrum disorder.

Members were eligible for study inclusion if they attended at least 3 sessions by July 1, 2025 (n=6632). Members were excluded from all analyses if they did not complete valid baseline and follow-up assessments for oppositional symptoms. Caregivers were excluded if they did not complete valid baseline assessments. The final analytic sample included 3781 members and 3780 caregivers (sample sizes for specific caregiver outcomes were as follows: n=3778 for stress, n=3779 for sleep, and n=3780 for burnout).

### Ethical Considerations

Before participating in care with Bend, eligible caregivers agreed to terms and conditions, and they consented to the treatment of a minor (on behalf of their child aged <18 years), agreeing to the collection of electronic data for the purposes of measurement-based care and for future data analyses. Adolescents (aged 13 to 17 years) also consented on behalf of themselves. Study participants were not recruited or compensated because all analyses were retrospective. Study procedures were approved by the Biomedical Research Alliance of New York (Study 23-12-034-1374; approved June 5, 2023).

### Treatment

Treatment with Bend has been described previously [28,34]. Caregivers enroll their child (aged 6 to 17 years) in care with Bend via the online web-based portal. During enrollment, caregivers provide information including insurance or employer coverage for services and demographic information for their child (date of birth, sex at birth, race and ethnicity, and gender identity). Then, they complete a series of MH screening questions followed by fully validated assessments to measure pediatric and caregiver MH symptoms (refer to the “Assessments” section). At the end of online enrollment, the caregiver schedules an intake session with a behavioral care manager (BCM), who is responsible for coordinating the member’s care between internal and external providers.

During the video-based intake session, the BCM meets with the member and their caregiver to discuss care options with Bend (eg, coaching and therapy), desired services, and insurance or employer coverage for services. Bend addresses pediatric MH challenges using a collaborative care approach, by which care may involve the following Bend providers: behavioral health coach (“coach”), licensed therapist (“therapist”), or psychiatric provider (nurse practitioner or psychiatrist). Based on the information gathered before and during intake, BCMs assign one or more of these providers to each member’s care team. All members are assigned a coach. Those who have more severe or complex MH symptoms are also assigned a therapist. Psychiatric providers are also assigned to a member’s care team if medication management (“psychiatry”) is indicated or included in the referral. Members and their caregivers regularly participate in video-based sessions with providers on their care team, with 30-minute coaching sessions typically scheduled twice monthly and 45-minute therapy sessions once monthly, as applicable. Psychiatry sessions are as needed. BCMs continue to coordinate care and monitor progress throughout participation with Bend.

Coaching and therapy sessions follow module-based care programs designed to address specific symptom domains—for example, ADHD, problematic behaviors, anxiety, and depression—using evidence-based methods delivered over approximately 12 weeks. Care programs are designed to be age-appropriate, with age-based variations between care plans for children (aged <13 years) and adolescents (aged 13 to 17 years). Care programs deliver caregiver interventions (eg, parent training) alongside member-targeted pediatric interventions. Caregiver-targeted interventions are a significant component of care for child programs (vs adolescent programs), as well as programs addressing symptoms known to be well-managed by parent involvement (eg, problematic behaviors). Between sessions, content from care programs is reinforced through an online learning resource center (including supplemental information and activities), and caregivers can communicate with their assigned care team using asynchronous messaging. To enable measurement-based care and monitor progress, members and caregivers complete follow-up MH assessments monthly.

### Assessments

Child and adolescent opposition, ADHD (inattention and hyperactivity), anxiety, depression, and sleep problems are initially screened by a series of brief MH screener questions. When symptoms are flagged by responses to the screeners (screen-in), symptoms are measured using full validated assessments. Caregivers of children and adolescents (aged <18 years) respond to questions for opposition and ADHD (inattention and hyperactivity), given the higher validity of proxy report for these symptom assessments [42]. Caregivers of children (aged <13 years) respond to the anxiety, depression, and sleep questions on behalf of their child. To facilitate accurate reporting and incorporate multiple perspectives, caregivers are instructed to complete assessments with their child, consulting on current behaviors and feelings. Adolescents (aged 13 to 17 years) self-report their own anxiety, depression, and sleep problems.

Opposition is screened by the question “...how much (or how often) has your child/teen had problematic behaviors in relation to others?” and inattention and hyperactivity are screened by the single inattention question from the *DSM-5* (*Diagnostic and Statistical Manual of Mental Disorders, Fifth Edition*) cross-cutting measure [3]. The *DSM-5* cross-cutting measure also screens child anxiety (3 items) and depression (2 items), and sleep problems for children and adolescents (1 item) [3]. Responses to the opposition and *DSM-5* cross-cutting screeners are on a 5-item Likert-type scale from “Not at all” (score=0) to “Nearly every day” (score=4). Adolescent anxiety and depression are screened using the first 2 questions from the Generalized Anxiety Disorder 7-item (GAD-7) and Patient Health Questionnaire 9-item (adolescent version; PHQ-9A) [43,44]. These measures are known as the Generalized Anxiety Disorder 2-item (GAD-2) and Patient Health Questionnaire 2-item (PHQ-2), respectively, and have been widely validated as screeners [45,46]. GAD-2 and PHQ-2 responses are on a 4-item Likert-type scale from “Not at all” (score=0) to “Nearly every day” (score=3).

Full assessments are completed based on responses to the MH screeners (screen-in; refer to Multimedia Appendix 1 for details). Opposition and ADHD symptoms are assessed using the Swanson Nolan and Pelham-IV (SNAP-IV), which includes 26 items total to measure opposition (items 19‐26), inattention (items 1‐9), and hyperactivity (items 10‐18) symptoms. Child anxiety (10 items) and depression (11 items), and child and adolescent sleep problems (8 items) are assessed using the corresponding Patient-Reported Outcomes Measurement Information System assessments [47-49], as recommended by the *DSM-5* for these age groups. Adolescent anxiety and depression are assessed using the remaining items from the GAD-7 and PHQ-9A [43,44], respectively. For the PHQ-9A, the final item regarding suicidal ideation is not presented due to the asynchronous delivery of the assessment [50]; thus, the measure is 8 items.

Caregivers self-report their own stress, burnout, and sleep. Caregiver stress is screened by 2 items from the 18-item Parental Stress Scale (PSS) [51], which measures distress related to caregiving responsibilities and the parent-child relationship. The PSS has been validated in clinical populations [52]. Caregivers complete the remaining 16 items of the PSS if stress is flagged by screeners [51]. For continuity between member-caregiver dyads, caregivers self-report the frequency of sleep problems in the past 2 weeks using the child and adolescent screening item [3]. Caregivers rate their subjective burnout using a validated single item with a 5-item Likert-type scale from “I enjoy my work. I have no symptoms of burnout” (score=1) to “I feel completely burned out...” (score=5) [53]. Details on all full assessments are in Multimedia Appendix 1.

### Measures

Member characteristics, clinical information, care engagement, and member and caregiver MH assessments were obtained from Bend’s electronic health records. The following member characteristics were used: age in years (at baseline assessment), sex (male, female, and nonbinary), gender (male, female, transgender, nonbinary, and other), and ethnicity (reported categories: White, Asian, Black or African descent, Hispanic or Latino, and Other). Diagnosed conditions were reported in the following categories: ADHD (any subtype), ODD or CD, anxiety disorders (eg, generalized anxiety disorder), and major depressive disorder or dysthymia. Assessment scores were aggregated per standard procedures for each validated assessment [42-44,47-49,53]. To facilitate clear reporting, severity categories (based on score) were reported as follows: not significant for screened out (ie, full assessment not taken) and low severities, subclinical for mild severity, and clinical for moderate and severe severities.

The baseline assessment was defined as the most recent completed within the 30 days before intake (preintake). If no preintake baseline was identified, the earliest assessment completed before the first session with a coach, therapist, or psychiatrist was retained as baseline. Assessments completed before baseline were excluded. All assessments after baseline were follow-up assessments. The timing of follow-up assessments was the date of completion relative to baseline, reported in 30-day months.

The first care program assigned was summarized as “externalizing” for the ADHD, executive functioning, and problematic behaviors care programs, and all other programs (eg, anxiety and resilience) were considered not externalizing. Total sessions were calculated as the aggregate sum of attended coaching, therapy, and psychiatry sessions within the study timeframe. Session counts were also calculated for each provider type. Sessions per month were calculated as total sessions divided by the duration in care (first to last session). For members with a duration in care of less than 1 month, the denominator of this calculation was set to 1 month to prevent artificial inflation of sessions per month values. Members were categorized as having therapy or psychiatry if they attended at least one session with the corresponding provider during the study period. Members were classified as having comorbid externalizing symptoms if they had clinical inattention or hyperactivity at baseline, and comorbid internalizing symptoms if they had clinical anxiety, depression, or sleep problems at baseline. Additional details on measures are in Multimedia Appendix 1.

### Statistical Analysis

Data preparation and statistical analysis were conducted using R (version 4.4.1; R Core Team) [54]. An alpha level of .05 was used for all statistical tests. Members were assigned oppositional severity groups (“group”) based on baseline severity: not significant, subclinical, or clinical. Group was coded numerically (ordered by severity) for analyses, as appropriate. Assessment scores were rescaled so that 0 represented the lowest possible score, as applicable. Data preparation methods are further detailed in Multimedia Appendix 1.

### Child and Adolescent Baseline Symptoms and Characteristics

The following characteristics were described by the oppositional severity group: age (in years; mean, SE), and the percentages of females and gender-sex conforming members, percent representation by ethnicity category, percentages of each type of MH diagnosis, percentages with clinical baseline MH symptoms (inattention, hyperactivity, anxiety, depression, and sleep problems). Treatment with Bend was described by the percentage of members with an externalizing symptom care program as their first care program, and those in therapy or psychiatry. Continuous variables were compared between groups using a linear model and categorical variables were compared using Cochran-Armitage trend tests with 2 levels. For ethnicity, levels were collapsed to White versus non-White; all other variables retained their original levels.

### Change in Oppositional Symptoms

Full-sample duration in care was described as median (IQR) months from first to last session, and median (IQR) months from baseline to follow-up assessments. Central trends (median [IQR]) for coaching, therapy, psychiatry, and total sessions attended were reported, with therapy and psychiatry sessions only reported for those in the corresponding type of care. Rates of oppositional symptom improvement (score decrease) and recovery (score <8) at each member’s last follow-up were reported for subclinical and clinical oppositional severity groups.

Changes in oppositional symptoms over the 7 months after baseline (refer to Multimedia Appendix 1 for details) were assessed using linear mixed-effects models of score over months (from baseline assessment). To determine the most accurate functional form of time, linear (months), quadratic (months^2^), and logarithmic (log [months+1]) models were evaluated. Each model included time, oppositional severity group, and their interaction as primary predictors, with a random effect of participant (member) on the intercept. Based on prior research for this program [35,55,56], we controlled for sex (female), age (in years), internalizing and externalizing symptoms, participation in an externalizing care program, therapy, psychiatry, and average sessions per month. All continuous predictors except time were mean-centered to reduce multicollinearity. Likelihood ratio tests (LRTs) identified the logarithmic model as the best fit. Adding a random effect of participant on the slope further improved model fit (*P*<.001) and was, therefore, retained. Finally, we evaluated potential moderators of symptom change by using LRTs to test interactions between time and each fixed effect. The final model included oppositional severity group, age (centered), externalizing care program, and psychiatry as interactions, and all other effects were retained as main effects.

Symptom change was evaluated at the full sample’s typical treatment duration using both observed and model-estimated values to characterize treatment responsiveness and to confirm model accuracy. Model-estimated scores for baseline and follow-up at 3.73 months (median follow-up) were generated for each group. Observed values were calculated using scores from baseline and final follow-up assessments completed within 4 months. Model-estimated and observed values were used to calculate the percentage reduction in symptoms from baseline to follow-up. Comprehensive details regarding oppositional model selection and refinement, including LRT results, are provided in Multimedia Appendix 1.

To prepare for caregiver symptom modeling, each child’s rate of improvement at the typical treatment duration was calculated as the estimated instantaneous slope at 3.73 months. Estimates were derived from member-specific fixed and random effects to isolate the rate of improvement from other covariates. The rate was subsequently inverted and mean-centered, such that higher values represent faster improvement.

### Caregiver Symptoms and Change in Oppositional Symptoms

Caregiver symptoms were first described as the percentage reporting stress, burnout, and sleep problems for all members and by oppositional severity group, with between-group differences tested by the Cochran-Armitage test. The change in likelihood for elevated caregiver symptoms associated with each 1-point increase in baseline oppositional symptom severity was expressed as odds ratios (ORs) with 95% CIs and associated *P* values.

For caregivers with mild-to-severe stress (n=2129), burnout (n=1680), and sleep problems (n=1760) at baseline, symptom severity (score) over the first 7 months of follow-up was assessed by linear mixed-effects models with fixed effects of time, oppositional symptom severity group, child’s rate of improvement (centered), and interactions of each effect with time. A random effect of participant (member) on the intercept accounted for individual differences. Months (log-transformed) were used as the time variable because logarithmic time was identified as the best-fit functional form for all caregiver models. Additionally, a random effect of participant (member) on the slope improved model fit and was retained in all models. To further describe associations between caregiver symptoms and child oppositional symptoms, modeled caregiver symptom trajectories were plotted by oppositional severity group and child treatment response (derived from rate of improvement), including slow responders (rate=–SD), typical responders (rate=0), and fast responders (rate =+SD).

## Results

### Child and Adolescent Baseline Symptoms and Characteristics

**Figure 1.**
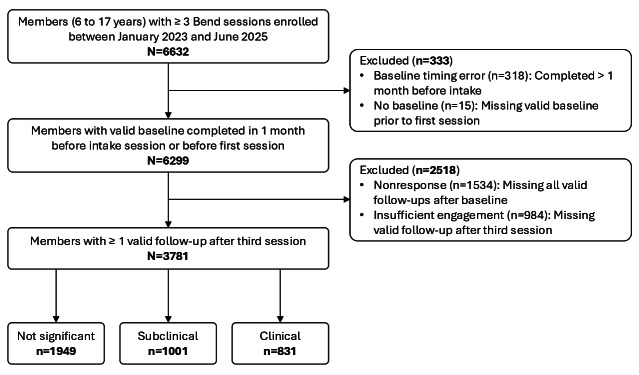
Participant study flow chart, including oppositional severity group assignments based on baseline symptoms.

Oppositional symptoms were evaluated in N=3781 children and adolescents (Figure 1). Given baseline symptoms, the oppositional severity group assignments were: 51.55% (1949/3781) not significant, 26.47% (1001/3781) subclinical, and 21.98% (831/3781) clinical (moderate: 13.96% [528/3781] and severe: 8.01% [303/3781]). Regarding characteristics (Table 1), children and adolescents with more severe symptoms tended to be younger (*P*<.001), were less likely to be female (*P*<.001), and more likely to be White (ethnicity: *P*<.001). They also had higher rates of ADHD, ODD, and anxiety disorder diagnosis, as well as elevated MH symptoms (except for anxiety; all *P*<.001). Externalizing symptom care programs and therapy were associated with more severe symptoms (all *P*<.001).

**Table 1.** Characteristics reported for children in each oppositional severity group^a^.

Characteristic	Not significant (1949/3781, 51.55%)	Subclinical (1001/3781, 26.47%)	Clinical (831/3781, 21.98%)	Between-group test; *t* test (*df*) or Z score	*P* value
Age (years), mean (SD)	11.77 (3.15)	10.78 (3.07)	10.39 (3.06)	–11.52 (3779)^b^	<.001^c^
Sex (female), n (%)	1089 (55.87)	461 (46.05)	363 (43.68)	6.48^d^	<.001^c^
Gender-sex conforming, n (%)	1877 (96.31)	979 (97.80)	805 (96.87)	1.21^d^	.23
Ethnicity, n (%)				3.50^d^	<.001^c^
White	969 (49.72)	519 (51.85)	475 (57.16)		
Asian	104 (5.34)	61 (6.09)	25 (3.01)		
Black or African descent	99 (5.08)	29 (2.90)	39 (4.69)		
Hispanic or Latino	108 (5.54)	42 (4.20)	36 (4.33)		
Other	669 (34.33)	350 (34.97)	256 (30.81)		
Diagnosed conditions, n (%)					
ADHD^e^	459 (23.55)	360 (35.96)	369 (44.40)	11.37^d^	<.001^c^
ODD^f^ or CD^g^	3 (0.15)	33 (3.3)	81 (9.75)	13.14^d^	<.001^c^
Anxiety disorder	1003 (51.46)	458 (45.75)	309 (37.18)	6.90^d^	<.001^c^
MDD^h^ or dysthymia	391 (20.06)	181 (18.08)	169 (20.34)	0.16^d^	.87
Clinical MH^i^ symptoms, n (%)					
Inattention	285 (14.62)	307 (30.67)	416 (50.06)	19.61^d^	<.001^c^
Hyperactivity	89 (4.57)	143 (14.29)	250 (30.08)	18.39^d^	<.001^c^
Anxiety	781 (40.07)	390 (38.96)	348 (41.88)	0.67^d^	.51
Depression	581 (29.81)	323 (32.27)	344 (41.40)	5.67^d^	<.001^c^
Sleep problems	717 (36.79)	417 (41.66)	399 (48.01)	5.57^d^	<.001^c^
Externalizing symptom care program, n (%)	416 (21.34)	349 (34.87)	391 (47.05)	13.^d^89	<.001^c^
Therapy, n (%)	830 (42.59)	476 (47.5)	401 (48.26)	3.08^d^	.002^j^
Psychiatry, n (%)	996 (51.10)	540 (54.15)	450 (54.15)	1.67^d^	.10

a Between-group test (*t* test and Cochran-Armitage tests) results identify differences in characteristics between oppositional severity groups. Cochran-Armitage test results are reported for categorical variables, and *t* test results for age.

b *t* test value.

c *P*<.001.

d Z value.

e ADHD: attention-deficit hyperactivity disorder.

f ODD: oppositional defiant disorder.

g CD: conduct disorder.

h MDD: major depressive disorder.

i MH: mental health.

j *P*<.01.

### Change in Oppositional Symptoms

In the first 7 months, the first and last sessions were a median of 2.63 (IQR 1.33‐4.7) months apart, and final follow-up assessments were a median of 3.73 (IQR 2.33‐5.87) months after baseline. Members attended a median of 7 (IQR 4‐12) sessions, corresponding with an average of 3.00 (SD 0.82) sessions per month. Members attended 6 (IQR 3‐9) coaching sessions. Members with therapy (45.15%, 1707/3781) had a median of 3 (IQR 2‐4) therapy sessions, and members with psychiatry (52.53%, 1986/3781) had a median of 1 (IQR 1‐1) psychiatry session. At the last follow-up, 73.93% (740/1001) of the subclinical oppositional severity group improved and 52.55% (526/1001) recovered, while 82.43% (685/831) of the clinical severity group improved and 23.83% (198/831) recovered.

**Figure 2.**
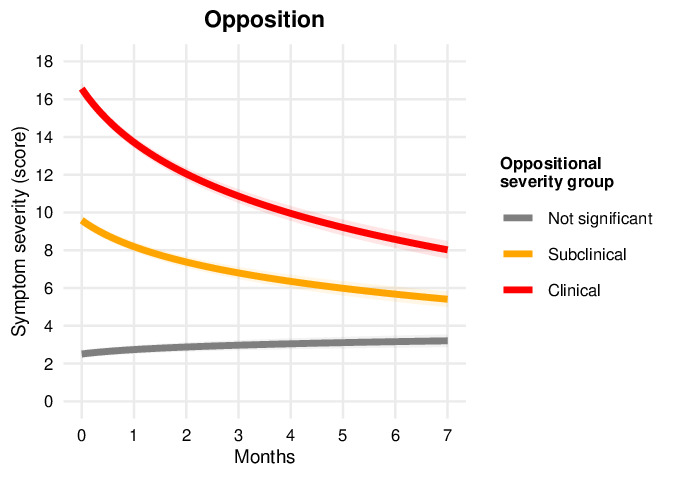
Logarithmic trends for oppositional symptoms during care for oppositional severity groups.

Symptom trajectories fit a logarithmic model, characterized by rapid initial improvement during the first several months of care followed by a gradual decline in monthly improvements. Full results from the logarithmic model are reported in Table 2. In brief, oppositional symptoms improved over months (log-transformed; *P*<.001). Symptoms were more severe for members with greater baseline severity (*P*<.001), and those with comorbid externalizing symptoms (*P*<.001), comorbid internalizing symptoms (*P*=.03), an externalizing care program (*P*<.001), therapy (*P*<.001), and greater sessions per month (*P*=.01). Symptoms were less severe for older members (*P*=.004) and females (*P*=.006). While psychiatry was associated with less severe symptoms, the main effect did not reach statistical significance (*P*=.07). Rates of symptom improvement were significantly greater (faster improvement) for members with more severe baseline symptoms (*P*_interaction_<.001). Specifically, those with clinical symptoms demonstrated rapid improvement over the first several months, whereas the subclinical group showed more gradual improvement. Scores for the not significant oppositional severity group remained largely stable (Figure 2). Rate of improvement was also significantly greater for older members (*P*_interaction_<.001) and significantly smaller for members with psychiatry (*P*=.01). At the sample’s typical final follow-up (3.73 months), the model-estimated aggregate symptom reduction was 27.73% for the subclinical oppositional severity group and 34.48% for the clinical oppositional severity group. These estimates were consistent with observed improvements across groups (Table 3).

**Table 2.** Results from the logarithmic model of oppositional symptom severity during care with the DMHI^a^.

Effect	Estimate (SE)	*t* test (*df*)	*P* value
Main effects			
Intercept	9.55 (0.11)	85.89 (17039)	<.001^b^
Months (log-transformed)	−1.92 (0.07)	−26.83 (17039)	<.001^b^
Oppositional severity group	9.93 (0.09)	112.65 (17039)	<.001^b^
Age at baseline	−0.04 (0.02)	−2.89 (17039)	.004^c^
Sex (female)	−0.24 (0.09)	−2.76 (17039)	.006^c^
Comorbid externalizing symptoms	0.83 (0.10)	8.04 (17039)	<.001^b^
Comorbid internalizing symptoms	0.21 (0.09)	2.27 (17039)	.02^d^
Externalizing care program	0.45 (0.11)	4.04 (17039)	<.001^b^
Therapy	0.37 (0.09)	3.94 (17039)	<.001^b^
Psychiatry	−0.17 (0.10)	−1.82 (17039)	.07
Sessions per month	0.13 (0.06)	2.31 (17039)	.02^d^
Interactions with months (log-transformed)			
Oppositional severity group	−3.14 (0.08)	−38.78 (17039)	<.001^b^
Age at baseline	−0.06 (0.01)	−4.20 (17039)	<.001^b^
Externalizing care program	0.17 (0.10)	1.67 (17039)	.10
Psychiatry	0.22 (0.09)	2.54 (17039)	.01

a DMHI: digital mental health intervention.

b *P*<.001.

c *P*<.01.

d *P*<.05.

**Table 3.** Model-estimated and observed baseline and follow-up scores^a^ at the median treatment duration, and the aggregate percent reduction from baseline to follow-up.

Oppositional severity group	Baseline score, mean (SD)	Follow-up score^a^, mean (SD)	Percent reduction (%)
Not significant (n=1949)			
Model-estimated	2.75 (1.14)	3.45 (2.76)	−25.12
Observed	2.56 (2.65)	3.35 (3.66)	−30.84
Subclinical (n=1001)			
Model-estimated	10.14 (1.21)	7.33 (2.96)	27.73
Observed	10.41 (1.67)	7.83 (4.33)	24.77
Clinical (n=831)			
Model-estimated	17.39 (1.66)	11.39 (4.01)	34.48
Observed	17.82 (3.11)	12.34 (5.73)	30.76

a Model-estimated values are derived from a logarithmic mixed-effects model and calculated at the sample’s median treatment duration (3.73 months). Observed values represent the last follow-up assessment completed within the first 4 months of care for each member.

### Caregiver Symptoms and Change in Oppositional Symptoms

At baseline, 57.34% (2129/3713) of caregivers reported stress, 45.14% (1680/3722) reported burnout, and 47.35% (1760/3717) reported sleep problems; these rates differed across oppositional severity groups (all *P*<.001; Table 4). As hypothesized, greater oppositional symptom severity was associated with significantly greater odds of elevated caregiver stress (OR 1.11, 95% CI 1.10-1.12), burnout (OR 1.06, 95% CI 1.05-1.08), and sleep problems (OR 1.03, 95% CI 1.02-1.04); all *P*<.001. Stress was the most strongly associated with oppositional symptoms, with a 10-point change in oppositional symptom score (eg, an increase from subclinical to clinical symptoms) associated with an 184% increase in odds of caregiver stress.

**Table 4.** Caregiver symptoms reported for each oppositional severity group^a^.

Symptom	Not significant, n/N (%)	Subclinical, n/N (%)	Clinical, n/N (%)	Between-group test (Z score)	*P* value
Stress	844/1914 (44.10)	634/980 (64.69)	651/819 (79.49)	17.91	<.001
Burnout	720/1919 (37.52)	469/980 (47.86)	491/823 (59.66)	10.86	<.001
Sleep problems	832/1918 (43.38)	496/978 (50.72)	432/821 (52.62)	4.87	<.001

a Between-group test (Cochran-Armitage tests) results identify differences in caregiver symptoms between oppositional severity groups.

**Figure 3.**
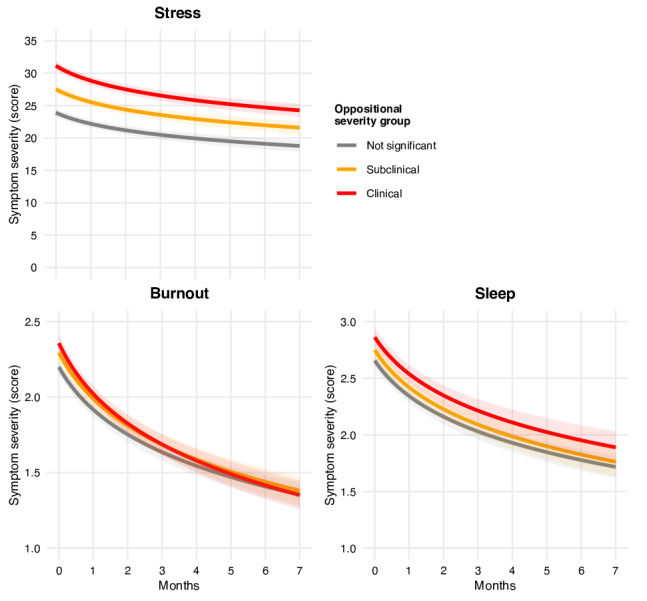
Longitudinal trajectories for caregiver symptoms over months, reported for each oppositional severity group.

**Figure 4.**
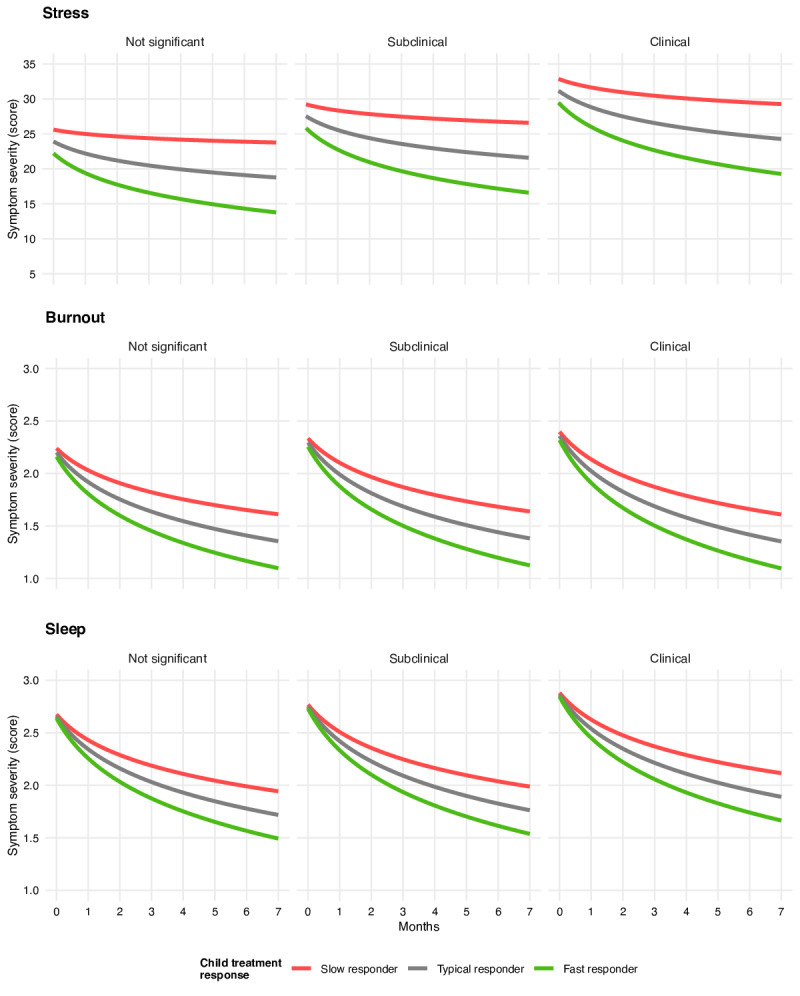
Longitudinal trajectories for caregiver symptoms over months, reported by child treatment response for each oppositional severity group.

All logarithmic model estimates for caregiver symptoms over months are reported in Table 5. Caregiver stress, burnout, and sleep problems decreased over months (all *P*<.001), following logarithmic curves with rapid improvement in the first several months and then more gradual declines later in care. More severe caregiver symptoms were associated with more severe oppositional severity (all *P*<.001). Faster improvement in child symptoms (greater child rate of improvement) was associated with less severe stress (*P*<.001) and burnout (*P*=.04), though this main effect was not significant for sleep (*P=*not significant). Regarding the magnitude of change in caregiver symptoms over time (Figure 3), more severe oppositional symptoms were associated with larger improvement in stress (*P*_interaction_<.001) and burnout (*P*_interaction_=.02), whereas this effect was not observed for sleep (*P*_interaction_=not significant). Faster improvement in child symptoms was associated with larger improvements in all caregiver symptoms over time (all *P*<.001), as demonstrated by diverging child treatment response trajectories for slow, typical, and fast responders (Figure 4).

**Table 5.** Results from logarithmic models of caregiver symptom severity during care with the DMHI^a^.

Fixed effect	Estimate (SE)	*t* test (*df*)	*P* value
Stress			
Intercept	27.53 (0.19)	141.52 (9434)	<.001^b^
Months (log-transformed)	−2.88 (0.12)	−23.71 (9434)	<.001^b^
Oppositional severity group	5.13 (0.33)	15.60 (9434)	<.001^c^
Child’s rate of improvement	−6.32 (0.72)	−8.83 (9434)	<.001^b^
Months (log-transformed) × oppositional severity group	−0.60±0.21	−2.89 (9434)	.004^c^
Months (log-transformed) × child’s rate of improvement	−5.89 (0.45)	−13.21 (9434)	<.001^b^
Burnout			
Intercept	2.28 (0.02)	140.34 (7545)	<.001^b^
Months (log-transformed)	−0.44 (0.01)	−31.40 (7545)	<.001^b^
Oppositional severity group	0.11 (0.03)	4.09 (7545)	<.001^b^
Child’s rate of improvement	−0.15 (0.06)	−2.49 (7545)	.01^d^
Months (log-transformed) × oppositional severity group	−0.05 (0.02)	−2.27 (7545)	.02^d^
Months (log-transformed) × child’s rate of improvement	−0.40 (0.05)	−7.58 (7545)	<.001^b^
Sleep problems			
Intercept	2.76 (0.02)	127.03 (7921)	<.001^b^
Months (log-transformed)	−0.46 (0.02)	−26.02 (7921)	<.001^b^
Oppositional severity group	0.15 (0.04)	3.96 (7921)	<.001^b^
Child’s rate of improvement	−0.07 (0.08)	−0.88 (7921)	.38
Months (log-transformed) × oppositional severity group	−0.01 (0.03)	−0.37 (7921)	.71
Months (log-transformed) × child’s rate of improvement	−0.39 (0.07)	−5.84 (7921)	<.001^b^

a DMHI: digital mental health intervention.

b *P*<.001.

c *P*<.01.

d *P*<.05.

## Discussion

### Principal Findings

This study characterized child and adolescent (aged 6 to 17 years) oppositional symptoms and associated caregiver outcomes within the real-world setting of a family-centered DMHI. Oppositional symptoms were associated with age, sex, and comorbid MH diagnoses and symptoms. Oppositional symptoms improved during DMHI participation, and those with more severe symptoms at baseline improved more rapidly. Older children and adolescents tended to improve more quickly than younger children and adolescents, and participation in psychiatry was associated with slower improvement. While more severe oppositional symptoms were associated with caregiver sleep problems, stress, and burnout, caregivers of those with more severe oppositional symptoms reported larger improvements in their own symptoms, and caregiver improvements were more rapid for those whose child’s rate of oppositional symptom improvement was greater (eg, fast responders).

This study described oppositional symptom presentation in children and adolescents receiving treatment from a DMHI and identified unique demographic factors associated with symptoms. Approximately 1 in 5 (22%) of children and adolescents had clinically elevated oppositional symptoms, yet only 10% of those with clinical symptoms had a formal diagnosis of ODD or CD. These findings are largely consistent with broader literature; while the global prevalence of ODD and CD is relatively low (<5%) [4], prevalence rates of 28% to 65% have been observed in clinical samples [6]. Boys had more severe symptoms than girls, consistent with higher ODD diagnostic rates among males [5,57,58]. Nonetheless, 45% of children and adolescents with oppositional symptoms were female. Considering that diagnostic tools and criteria are largely based on behaviors more common in males with ODD versus females (eg, physical vs relational aggression), these results demonstrate the need to monitor and address ODD in both males and females [59]. This study also found that oppositional symptoms were less severe for older children and adolescents, likely reflecting the gradual decrease in the severity of oppositional symptoms (especially defiant behaviors) from preadolescence and into adulthood [9,60]. Those with more severe oppositional symptoms were more likely to have additional MH challenges, with inattentive and hyperactivity symptoms, and ADHD and ODD diagnoses particularly prevalent among those with problematic behaviors. This finding is expected given the high rate of comorbidity between ADHD and ODD [12,58]. Notably, however, children and adolescents with oppositional symptoms varied in their individual and clinical characteristics (eg, age, sex, and co-occurring MH conditions). The DMHI evaluated here implements measurement-based care using a standardized screening protocol, potentially flagging relevant clinical symptoms in otherwise undiagnosed youth and circumventing demographic-based biases by applying the same criteria across all participants. Ultimately, findings demonstrate the value of standardized and routine screening (such as Bend) to evaluate and address oppositional behaviors in the context of pediatric DMHIs.

Though most children and adolescents did not receive care through a targeted oppositional behavior track, Bend successfully reduced oppositional symptoms in the majority (78%) of those with clinical and subclinical presentations. Symptom reductions were evident monthly, with the largest improvements in the first months of care. This finding aligns with the broader literature demonstrating that oppositional symptom improvement is associated with participation in a variety of evidence-based behavioral programs, particularly those emphasizing parenting and caregiver interventions [1,23,24,61]. With Bend, children and adolescents who started with more severe oppositional symptoms improved more quickly over the course of care, suggesting that treatment through DMHIs like Bend is effective even for participants with more serious behavior problems. Other commercial DMHIs have demonstrated improvements across a range of pediatric symptoms with coaching and therapy [37,38], and several studies have shown reduction of MH symptoms, including anxiety, depression, and ADHD, during participation with Bend [28,34,35,56]. While there is evidence that digital game-like interventions may also address ADHD and ODD in children and adolescents [62-64], this is the first rigorous study, to our knowledge, to report significant improvements in oppositional symptoms via family-centered coaching and therapy with a DMHI.

While engagement with Bend’s DMHI was associated with substantial reductions in oppositional behaviors, there were differences in treatment responsiveness. First, older children and adolescents improved more quickly during care. Unlike younger children, older children and adolescents can take an active role in their treatment, potentially yielding greater gains from child-targeted interventions (eg, CBT and social problem-solving skill training) [24-27]. Additionally, because caregivers of older children are less likely to have elevated parental stress and burnout [22,40], they may be better equipped to support their child’s treatment (eg, via implementation of caregiver-directed interventions) [65,66]. Children and adolescents with psychiatry, on the other hand, tended to improve less quickly than those without psychiatry. This finding suggests that children and adolescents in psychiatry were less responsive to coaching or therapy alone, such as those with more complex cases and comorbidities. Thus, it is likely that medication management in psychiatry was appropriately assigned to those requiring this higher level of care. Notably, children and adolescents with more severe symptoms had more frequent sessions during care, but sessions per month were not a significant predictor of the rate of improvement, suggesting that care intensity was scaled to meet higher clinical need. This finding contrasts with other studies reporting associations between engagement metrics and better MH outcomes, including management of behavior problems [67]. Taken together, it is likely that factors beyond engagement—including demographics, caregiver involvement, and type of care—have stronger associations with symptom progress. Overall, DMHIs that have an individually tailored and family-centered approach may be an effective solution for treating opposition, even for those with severe and complex symptom presentation.

Bend’s family-centered approach provides simultaneous support to both pediatric members and their caregivers. Specifically, caregivers whose children were more responsive to care (eg, improved more quickly) also reported larger improvements in their own stress, burnout, and sleep. These findings are consistent with prior research highlighting the reciprocal relationship between child behavioral dysregulation and caregiver well-being, in which elevated externalizing symptoms in children and adolescents contribute to greater emotional strain, physiological stress, and impaired functioning in caregivers [14,15,17]. On the other hand, improvements in child behavior lessen caregiver burden and may have positive downstream effects on caregiver well-being [22,41,68]. While caregiver stress, burnout, and sleep problems improved over time in care, those supporting children with more severe behavior problems showed the largest gains. While other studies have demonstrated improvements in caregiver symptoms during pediatric DMHI treatment [21,22,39,40]—including a recent study showing co-occurring improvements in general pediatric symptoms and caregiver symptoms—this is the first to demonstrate the sensitivity of caregiver symptoms to their child’s oppositional behaviors. Indeed, reductions in caregiver stress and sleep problems are associated with better long-term outcomes, including lower incidence of MH problems, improved cardiovascular health, lower morbidity, and improved cognitive function [69-71]. These results position DMHIs such as Bend to support scalable, system-level change in pediatric and family MH care. Future studies should examine the effectiveness of opposition-specific care programs, as well as long-term outcomes of children and adolescents with symptoms of ODD.

### Strengths and Limitations

This study has several notable strengths. First, this study addresses significant research questions by combining pediatric and caregiver data, contributing greatly to the clinical and scientific knowledge base on the detection and treatment of ODD symptoms. Second, by conducting primary analyses on a large sample of member-caregiver pairs (N=3781) who meaningfully engaged with the DMHI in a real-world setting for at least 3 sessions, the findings presented are more reliable and generalizable than the previous study of oppositional symptoms with Bend [28]. Third, children and adolescents with subclinical and nonsignificant symptoms were included in analyses of change. Thus, findings highlight early intervention effects and the robustness of the DMHI and analytic strategy in detecting improvements among less symptomatic children. This is notable given that such individuals typically have smaller gains and are vulnerable to potential floor effects. Additionally, by measuring symptoms over care for those with nonsignificant symptoms at baseline, we demonstrate that few children and adolescents developed new oppositional symptoms during participation with the DMHI. Thus, we can be confident that our findings reflect substantive clinical change rather than a regression to the mean. Fourth, observed and model-estimated values for oppositional symptom improvement were comparable, demonstrating the generalizability and accuracy of the results reported here. The rigor of the study is further strengthened through the use of validated, psychometrically sound measures administered at repeated time points.

Regarding limitations of the study, its retrospective and observational design precludes causal inferences; observed improvements in caregiver outcomes may reflect co-occurring changes or shared contextual factors rather than direct effects of the DMHI. Indeed, we cannot determine the directionality of the relationship between pediatric and caregiver symptom changes. The findings presented here are specific to a single DMHI (Bend) and may not generalize to other digital platforms or traditional therapy. Further research should be conducted on different DMHI tools to pinpoint particularly effective interventions and determine whether findings are generalizable. This study represents a modest follow-up period of 7 months maximum, with a typical length of participation of four months. Prior research has shown mixed long-term outcomes for ODD symptoms, with some reporting diagnostic instability (particularly in younger children) [29,72,73] and others demonstrating sustained reductions in disruptive behaviors following intervention [74]. Future studies would benefit from an extended follow-up period to evaluate the long-term stability of symptom improvements. Regarding outcome measures, the SNAP-IV is a widely used tool for assessing ODD symptoms, but it is not intended for diagnostic purposes. While multiple screening tools exist, there is no consensus on best practice [24]. Nonetheless, the cut-off scores used in this study align with standardized guidelines [75]. Another limitation is the use of caregiver report for many pediatric symptoms. Caregiver report is broadly used to measure observable behaviors (including opposition and defiance), as well as symptoms in young children (eg, aged <13 years) [47]. However, outcomes may differ significantly by informant, particularly for internalizing symptoms and symptoms or behaviors that lack cross-situational consistency (eg, behaviors present at school and not at home) [76,77]. While caregivers in this study were encouraged to seek input from their child when reporting symptoms and behaviors, future studies should use a multi-informant framework. This approach would account for potential informant discrepancies and offer a more nuanced, comprehensive assessment of child functioning. Finally, though the models controlled for several variables, residual confounding from unmeasured factors may remain. For example, the study did not evaluate several critical child and caregiver characteristics—including pediatric health outcomes, caregiver MH history and demographics, and family socioeconomic circumstances—which are likely to influence outcomes [78-81]. These additional variables should be included in future research to better elucidate their impact on family-wide MH.

### Conclusions

This study provides novel evidence that oppositional symptoms in children and adolescents can improve meaningfully during participation in a family-centered DMHI, even in the absence of targeted ODD treatment. Improvements were observed across clinical and subclinical severity levels and were more pronounced in younger children and adolescents and those with more severe baseline symptoms. While engagement varied by symptom severity, session frequency did not predict the rate of improvement, suggesting that the DMHI may have effectively tailored care intensity to individual needs. Notably, improvements in caregiver outcomes were closely linked to child oppositional symptoms at baseline and during care, with greater oppositional severity and larger improvements associated with larger reductions in stress, burnout, and sleep problems. These findings underscore the promise of family-centered DMHIs (such as Bend) in delivering scalable, flexible, and family-centered care that supports both pediatric MH and caregiver well-being. Future work should examine the long-term stability of these outcomes, explore potential mechanisms of change, and evaluate how caregiver-level factors influence treatment responsiveness within digital care models.

## Supplementary material

10.2196/82039 Multimedia Appendix 1 Supplemental methods, including additional information on assessments and measures, and detailed methods for data processing and statistical analyses.

## Abbreviations

ADHD: attention-deficit hyperactivity disorder
BCM: behavioral care manager
CBT: cognitive behavioral therapy
CD: conduct disorder
DMHI: digital mental health intervention
*DSM-5*: *Diagnostic and Statistical Manual of Mental Disorders, Fifth Edition*
GAD-2: Generalized Anxiety Disorder 2-item
GAD-7: Generalized Anxiety Disorder 7-item
LTR: likelihood ratio test
MH: mental health
ODD: oppositional defiant disorder
OR: odds ratio
PHQ-2: Patient Health Questionnaire 2-item
PHQ-9A: Patient Health Questionnaire-9 adolescent version
PSS: Parental Stress Scale
SNAP-IV: Swanson Nolan and Pelham-IV
