# Supplementary material for: Family-Focused Digital Mental Health Care for Pediatric Oppositional Symptoms and Caregiver Outcomes: Retrospective Analysis
Source: JMIR Pediatr Parent. 2026 Jun 22;9:e82039. doi: 10.2196/82039 (PMC13286078; doi:10.2196/82039)
Supplement: Multimedia Appendix 1 [file pediatrics-v9-e82039-s001.docx]

# Methods

## Assessments

For child and adolescent opposition, inattention, and hyperactivity, a response of “Rare, less than a day or two” or more frequently to the corresponding screener question (single question for inattention and hyperactivity) prompts the full assessment for that symptom (screen-in); these assessments are a subset of questions from the Swanson, Nolan, and Pelham Rating Scale (SNAP-IV) [1]. The SNAP-IV includes a total of 26-items, including subsets of questions for inattention (items 1-9), hyperactivity (items 10-18), and opposition (items 19-26). Each item asks about behaviors in the past month. The opposition items include questions about losing temper, arguing, and defiance or refusal to adult requests. The inattention items include questions about difficulty sustaining attention and distractibility. The hyperactivity items include questions about fidgeting and interrupting or intruding on others. Responses to all SNAP-IV items are made on a 4-point Likert-type scale from “Not at all” (score = 0) to “Very much” (score = 3). Before April 1st, 2024, the inattention screener screened for both the inattention and hyperactivity full assessments, and the opposition screener question screened for only the opposition full assessment. From April 1st, 2024, onward, a positive response to either the inattention or opposition screener questions screened-in for the full assessments for inattention, hyperactivity, and opposition (i.e., triggered the full 26-item SNAP-IV assessment).

For child anxiety, child depression, and child sleep, a response of “Several days” or more frequently (score ≥ 2) to any of the corresponding screener questions triggers the corresponding Patient-Reported Outcomes Measurement Information System (PROMIS) assessment [2–4]. These assessments inquire about the frequency of typical anxiety symptoms (10-items), depression symptoms (11-items), and sleep problems (8-items) in the past seven (7) days. The anxiety assessment includes questions about feeling nervous, scared, and worried. The depression assessment includes questions about feeling sad, lonely, and stressed. The sleep assessment includes questions about restless sleep, sleep satisfaction, and trouble staying asleep. Responses to all items on the PROMIS anxiety and depression assessments are on a 5-point Likert-type scale from “Never” (score = 1) to “Almost Always” (score = 5). PROMIS sleep assessment responses are on various 5-point Likert-type scales that align with each question; some of these scales are reversed because positive sleep characteristics are measured (e.g., satisfaction with sleep).

For adolescent anxiety and depression, the adolescent screens-in to the full Generalized Anxiety Disorder 7-item assessment (GAD-7) and first 8-items of the Patient Health Questionnaire 9-item assessment (adolescent version; PHQ-9A) if the sum of responses to their respective 2-item screeners is ≥ 2 [5–8]. The GAD-7 and PHQ-9A inquire about the frequency of typical anxiety and depression symptoms, including worrying and trouble relaxing for anxiety, and having little interest in doing things and feeling bad about oneself for depression. The GAD-7 asks about feelings and behaviors in the past two (2) weeks, whereas the PHQ-9A is about the past seven (7) days. Responses to items on both scales—including the screener items—are made on 4-item Likert-type scales from “Not at all” (score = 0) to “Nearly every day” (score = 3). The adolescent sleep screener and full assessment are identical to the child sleep screener and full assessment, but with each item phrased as self-report versus parent-report [4]. For example, “Your sleep was restless.”

For caregiver stress, a response of “Undecided” or greater agreement (score ≥ 3) to either screener question from the Parental Stress Scale (PSS) triggers the remaining 16-items from the PSS [9]. Half of the items of the PSS are about negative parenting experiences—including difficulty balancing responsibilities and feeling overwhelmed—and the other half are about the positive and enjoyable aspects of parenting. Responses to all items are on a 5-point Likert-type scale ranging from “Strongly disagree” (score = 1) to “Strongly agree” (score = 5). For questions about positive parenting experiences, the item score is reversed such that higher agreement corresponds with a lower score. Burnout is measured by a single item about subjective work-related burnout [10], with responses from “I enjoy my work. I have no symptoms of burnout.” (score = 1) to “I feel completely burned out…” (score = 5).

Before January 23rd, 2024, all screeners were given each month, which then determined which full assessments were screened-in for that date. From January 23rd to August 9th, 2024, the full assessment was automatically screened-in for symptoms that had screened-in during a previous assessment, and screeners were only completed for symptoms that did not have previous screen-in. For example, if a child screened-in to take the opposition assessment at baseline, their follow-up assessments would not include the screeners and they would automatically be given the full assessment. Starting August 9th, 2024, all screeners were repeated each month and then full assessments were automatically screened-in for symptoms that had been screened-in during a previous assessment. For all assessments during the study period, responses to the screeners could trigger the full assessment (screen-in) regardless of historical symptoms. Screened-out and low symptom severity were both considered “not significant” to account for differences in the screening methods over time.

## Measures

Child and adolescent opposition, inattention, and hyperactivity symptom severity was calculated from aggregate scores on the corresponding subset of SNAP-IV symptoms, and then matched to symptom severity category based on score [1]. For opposition, the severity categories were: low (score < 8), mild (score 8-13), moderate (score 14-18), and severe (score > 18). For inattention and hyperactivity, the severity categories were: low (score < 13), mild (score 14-17), moderate (score 18-22), and severe (score > 22). Child anxiety and depression, and child and adolescent sleep symptom severity was calculated from aggregate scores on the corresponding PROMIS assessments, which were converted to T-Score and then matched to symptom severity based on T-score [2–4]: low (T-score < 55), mild (T-score 55-59.9), moderate (T-score 66.0-69.9), and severe (T-score ≥ 70.0). For adolescent anxiety, aggregate score was matched to symptom severity category [5]: low (score < 5), mild (score 5-9), moderate (score 10-14), severe (score 15-21). For adolescent depression, aggregate score was adjusted to account for the omitted ninth item (aggregate score X 9/8), and then adjusted score was matched to symptom severity category [6]: low (score < 5), mild (score 5-9), moderate (score 10-14), moderately severe (score 15-19), severe (score 20-27). For caregiver sleep, score was matched to severity per the DSM-5 cross cutting measure [4]: low (none or slight; score < 2), mild (score = 2), moderate (score = 3), severe (score = 4). For caregiver stress, aggregate score was matched to symptom severity categories using the methods described by others [9,11]: low (score < 18), mild (score 18-41), moderate (42-65), and severe (score > 65). Caregiver burnout severity was as follows [10]: low (score < 3), mild (score = 3), moderate (score = 4), and severe (score = 5). For all analyses, scores were scaled such that 0 was equivalent to the lowest measure score, and a score of 0 was assigned if the full assessment was not completed due to screening-out.

## Data preparation

The analytic sample for oppositional symptoms was restricted to valid assessments meeting two sequential criteria: 1) baseline assessment or follow-up assessment completed after at least one attended session since the previous assessment, and 2) follow-up assessment completed within two months of the previous assessment.

To ensure oppositional symptom analyses reflected accurate baseline measurement and engagement in care, member-level exclusions were applied sequentially: 1) n=333 excluded due to no valid baseline, including n=318 who completed baseline > 1 month prior to intake, and n=15 who were missing baseline before first session, and 2) n=2,519 excluded for lacking a valid follow-up after the 3rd session, including n=1,534 removed for missing all valid follow-ups after baseline, and n=984 removed for missing a valid follow-up after 3rd session. Additionally, to minimize bias from outliers with extended treatment durations, we restricted follow-up assessments to those completed in the first seven months (capturing the final follow-up for 80% [n=3,043] of the final analytic sample). Ultimately, n=3,781 members were retained in the final analyses, with 17,056 oppositional assessments (mean = 4.51 ± 1.77 assessments per member).

For the additional child and adolescent symptoms, where members in the final sample were missing a baseline assessment within the standard window, the nearest assessment to intake was retained as baseline: n=1 for hyperactivity and inattention, n=9 for anxiety, n=10 for depression, and n=4 for sleep problems. Finally, for caregiver symptoms, the same filtering procedures as used for oppositional symptoms were applied to baseline and follow-up assessments. Caregivers were excluded from all analyses if they lacked a valid baseline assessment, and caregivers were excluded from longitudinal modeling if they lacked a valid follow-up after the 3rd session. Final caregiver sample sizes were: n=3,778 for baseline stress and n=3,675 for longitudinal stress, n=3,779 for baseline sleep problems and n=3,670 for longitudinal sleep problems, and n=3,780 for baseline burnout and n=3,672 for longitudinal burnout. A total of 50,322 caregiver assessments were included in analyses.

## Statistical analysis

Linear mixed-effect models were fit using the bobyqa optimizer with maxfun = 2e5 to improve convergence stability, given the complexity of the models, selected based on diagnostic comparisons across optimizers. For modeling of oppositional symptoms over time, linear (months), quadratic (months^2^), and logarithmic (log[months + 1]) models were compared, and the logarithmic model was selected because it yielded the lowest Information Criteria values (AIC = 90,318; BIC = 90,442), compared to the linear (AIC = 90,919; BIC = 91,043) and quadratic models (AIC = 90,360; BIC = 90,507). Addition of a random effect of subject (member) on the slope yielded even lower Information Criteria values (AIC = 89,169; BIC = 89,308), and was retained in the model.

Each covariate was tested as an added interaction term to determine whether any modified symptom improvement over time. These additional models were compared to the logarithmic model (i.e., with covariates included as main effects) using likelihood ratio test (LRT). For any statistically significant LRT, the interaction of interest was added to the primary model. Age (P<.001), prescriber (*P*=.006), externalizing care program (*P*=.003) were retained as interactions. The following were not retained as interactions: sex (*P=*.66), externalizing symptoms (*P=*.12), internalizing symptoms (*P=*.49), therapy (*P=*.48), and sessions per month (*P=*.68). Generalized variance inflation factors (GVIFs) were used to evaluate multicollinearity, with all values < 5, indicating no concerning correlations. Generalized VIFs for the final model are reported in Table S1.

Table S1. Generalized VIFs for the final model of oppositional symptom severity during care with the DMHI.

| **Effect** | **GVIF** | **DF** | **GVIF^1/(2DF)^** |
| --- | --- | --- | --- |
| **Main effects** | | | |
| Months (log-transformed) | 2.76 | 1 | 1.66 |
| Oppositional severity group | 1.57 | 2 | 1.12 |
| Age at baseline (centered) | 1.30 | 1 | 1.14 |
| Sex (female) | 1.08 | 1 | 1.04 |
| Comorbid externalizing symptoms | 1.27 | 1 | 1.13 |
| Comorbid internalizing symptoms | 1.18 | 1 | 1.09 |
| Externalizing care program | 1.49 | 1 | 1.22 |
| Therapy | 1.24 | 1 | 1.11 |
| Psychiatry | 1.31 | 1 | 1.14 |
| Sessions per month (centered) | 1.24 |  | 1.11 |
| **Interaction with months (log-transformed)** | | | |
| x Oppositional severity group | 1.59 | 2 | 1.12 |
| x Age at baseline (centered) | 1.25 | 1 | 1.12 |
| x Externalizing care program | 1.79 | 1 | 1.34 |
| x Psychiatry | 2.32 | 1 | 1.52 |

GVIF: Generalized variance inflation factor

# References

1. Swanson JM, Sandman CA, Deutsch C, Baren M. Methylphenidate hydrochloride given with or before breakfast: I. Behavioral, cognitive, and electrophysiologic effects. Pediatrics 1983 Jul;72(1):49–55. PMID:6866591

2. Irwin DE, Gross HE, Stucky BD, Thissen D, DeWitt EM, Lai JS, Amtmann D, Khastou L, Varni JW, DeWalt DA. Development of six PROMIS pediatrics proxy-report item banks. Health Qual Life Outcomes 2012 Feb 22;10:22. PMID:22357192

3. Sherlock P, Blackwell CK, Kallen MA, Lai J-S, Cella D, Krogh-Jespersen S, Luby JL, Buss KA, Burns J, Wakschlag LS. Measuring PROMIS® Emotional Distress in Early Childhood. J Pediatr Psychol 2022 May 13;47(5):547–558. PMID:35552432

4. Forrest CB, Meltzer LJ, Marcus CL, De La Motte A, Kratchman A, Buysse DJ, Pilkonis PA, Becker BD, Bevans KB. Development and validation of the PROMIS Pediatric Sleep Disturbance and Sleep-Related Impairment item banks. Sleep Oxford University Press US; 2018;41(6):zsy054.

5. Spitzer RL, Kroenke K, Williams JBW, Löwe B. A brief measure for assessing generalized anxiety disorder: the GAD-7. Arch Intern Med 2006 May 22;166(10):1092–1097. PMID:16717171

6. Kroenke K, Spitzer RL, Williams JB. The PHQ-9: validity of a brief depression severity measure. J Gen Intern Med 2001 Sep;16(9):606–613. PMID:11556941

7. Plummer F, Manea L, Trepel D, McMillan D. Screening for anxiety disorders with the GAD-7 and GAD-2: a systematic review and diagnostic metaanalysis. Gen Hosp Psychiatry 2016;39:24–31. PMID:26719105

8. Kroenke K, Spitzer RL, Williams JBW. The Patient Health Questionnaire-2: validity of a two-item depression screener. Med Care 2003 Nov;41(11):1284–1292. PMID:14583691

9. Berry JO, Jones WH. The Parental Stress Scale: Initial Psychometric Evidence. J Soc Pers Relatsh 1995 Aug;12(3):463–472. doi: 10.1177/0265407595123009

10. Mateo-Rodríguez I, Knox E, Oliver-Hernandez C, Daponte-Codina A. Validation of a Single-Item Screening Measure of Burnout in a Sample of Spanish Health Workers. Soc Sci Multidisciplinary Digital Publishing Institute; 2023 Oct;12(10):546. doi: 10.3390/socsci12100546

11. Zelman JJ, Ferro MA. The Parental Stress Scale: Psychometric Properties in Families of Children With Chronic Health Conditions. Fam Relat 2018;67(2):240–252. doi: 10.1111/fare.12306
